# Physical and cognitive characteristics of a group exercise program for children treated for brain tumors

**DOI:** 10.3389/fresc.2025.1709309

**Published:** 2026-01-15

**Authors:** Jennifer L. Ryan, Krista Johnston, Éric Bouffet, Ute Bartels, Brian W. Timmons, Cynthia B. de Medeiros, Donald J. Mabbott

**Affiliations:** 1 Neurosciences and Mental Health Program, Research Institute, The Hospital for Sick Children, Toronto, ON, Canada; 2 Divisions of Hematology/Oncology, The Hospital for Sick Children, Toronto, ON, Canada; 3 Department of Pediatrics, Child Health and Exercise Medicine Program, McMaster University, Hamilton, ON, Canada; 4 Department of Psychology, University of Toronto, Toronto, ON, Canada

**Keywords:** brain tumor, cardiovascular, children, coordination, exercise, object control

## Abstract

**Background:**

Children treated for brain tumor (CTBT) experience lasting physical and cognitive impairments that impact quality of life. Given the pervasive impact of brain tumor treatments on cognition, we designed a group exercise program with the specific goal of improving cognition in CTBT. A feasibility study evaluating the program demonstrated improved cognitive and physical outcomes. However, the exercises varied across sessions to maximize participant engagement throughout the 12-week program, which made it difficult to describe the program contents and identify how each element contributed to the observed improvements.

**Objective:**

As a precursor to identifying the effective elements within the group exercise program for CTBT, this study characterized the content of our program according to the exercise categories observed in the exercise sessions, the physical fitness components within the exercises, and the cognitive demand of the exercises.

**Methods:**

This retrospective descriptive analysis coded the content of 50 videos from the exercise program sessions by exercise category (warm-up, aerobic training, games, sports, cool-down), physical fitness components (cardiovascular, coordination, speed, agility, object control, strength, balance, flexibility), and cognitive demand (low, medium, high). Data were descriptively summarized.

**Results:**

Most of the exercise session was spent on games, followed closely by sports. 33% of the exercise session involved exercises with high amounts of cardiovascular content and 46% of the exercise session involved exercises with moderate amounts of cardiovascular content. Exercises with high cognitive demand had the most coordination and object control content. Exercises with medium cognitive demand had the most cardiovascular content.

**Conclusion:**

The presence of exercises with either high cardiovascular content or high coordination was the hallmark of our group exercise program for CTBT. These features should be manipulated in future exercise program evaluations to determine their impact on cognitive and physical outcomes in CTBT.

## 1 Introduction

Children and youth treated for brain tumors (CTBT) contend with cognitive and physical sequelae that negatively affect their quality of life (1–3). While their long-term physical limitations (fatigue, and decreased balance, strength, coordination, agility, and walking/running speed) can limit their participation in physical activity (1, 3–5), CTBT' cognitive impairments (decreased processing speed, working memory, attention, problem solving, visual motor control, and visuospatial skills) have an even greater impact on daily life, as these impairments negatively affect their academic, social, and emotional well-being (1, 6–8). Fortunately, exercise has the strong potential to improve both cognitive and physical function in CTBT (9, 10). Due to the positive impact that aerobic exercise has had on cognition in typically developing (11, 12) and other clinical populations (13–15), our lab conducted a 12-week group exercise program feasibility trial for CTBT with the primary aim of improving cognition (16). The program allowed instructors to vary the exercises within a framework of exercise categories to maximize participation with the goal of maintaining participants' heart rates at 80% of their maximum for at least 30 min of each session (16). While the feasibility trial demonstrated preliminary improvements in coordination, physical fitness, and reaction time during cognitive testing in 16 CTBT (16), we identified the need to better understand the content of the exercise program as a precursor to determining how the physical and cognitive elements within the program contributed to study outcomes. To address these concerns, this study will document and analyze the exercise session content of videos from our 12-week group exercise program for CTBT according to common characteristics that can be applied across exercises.

Exercise is a subset of physical activity that involves structured, repetitive, and purposeful body movement with the goal of improving physical fitness (17). Physical fitness consists of: (1) health-related fitness components, such as cardiorespiratory endurance, muscular endurance, muscular strength, body composition, flexibility, and (2) skill-related fitness components, such as agility, balance, coordination, speed, power, reaction time (17–21). The presence of these components varies depending on the activities within an exercise program and likely influences the aspects of physical fitness that improve with program participation. Physical fitness is also associated with cognitive function and brain health (18), which has led to the increased use of exercise interventions to target cognitive outcomes. Aerobic/cardiovascular exercise has been of particular interest in clinical and non-clinical populations (22–24) as it is thought to improve cognitive function by increasing the concentration of brain-derived neurotrophic factor (25) and growth factors (e.g., insulin growth factor) that promote neuroplasticity (26). Additionally, aerobic exercise is associated with increased hippocampal volume, which plays a role in memory and learning (16, 27–29).

Exercise also moderates cognitive function through the cognitive resources required to execute complex motor skills and the cognitive demands of goal-directed exercise (30, 31). When a motor skill involves more complex cognitive processes, there is increased activation of the brain regions involved in these higher order cognitive processes (30), and repeated practice of these skills improves executive function in children (20, 21, 32, 33). Within goal-directed exercise, the cognitive demands differ depending on whether the activity is self-paced or externally paced and individual or team-based, as these variations require different levels of preparation, attention, and responses (34, 35). Self-paced exercises allow the participant time to prepare and execute the exercise, whereas externally paced activities require rapid decision-making and reactions (35). Team-based sports have an added layer of cognitive demand over individual activities because they not only require participants to react to the actions of their opponents, but they also require the participants to anticipate and coordinate their actions with their teammates (34).

Documenting our exercise program content according to the physical and cognitive factors within the exercises that may influence intervention outcomes provides a common ground for detailing an intervention that involves a variety of exercises over the intervention period, and allows us to explore the intersection of these cognitive and physical factors. This approach to reporting intervention content also permits accurate replication of the intervention and promotes treatment fidelity in clinical trials and clinical practice. This study documents and analyzes the exercise program content from our feasibility trial for CTBT according to the exercise categories observed in the exercise sessions (objective 1), the physical fitness components within the exercises (objective 2), and the cognitive demand of the exercises (objective 3). While the purpose of this study was to describe the exercise session content, we anticipated that cardiovascular content would be present in most exercises, and the exercises would have a range of cognitive demand ratings based on the format of the exercise program.

## 2 Methods

### 2.1 Study design

This study used a retrospective, descriptive design to explore the content of a group exercise program for CTBT with the aim of generating hypotheses about the link between physical and cognitive content of the exercise sessions, rather than providing confirmatory evidence of their association.

### 2.2 Participants

We conducted a descriptive content analysis of exercise session videos from our completed group exercise feasibility trial for CTBT (ClinicalTrials.gov NCT01944761) that involved children/youth (6–17 years, 11 months of age at study enrollment) who were: diagnosed with a brain tumour, 1–15 years between diagnosis and study enrollment, medically stable (i.e., in remission), capable of complying with study instructions, and safe to participate in an exercise program (16). Research ethics approval was obtained from the Research Ethics Board at The Hospital for Sick Children (Toronto, Canada) (#1000019124). Informed consent was obtained for study participation. Videos of the exercise sessions were recorded as part of data collection in the study, with 54 exercise videos available across two study cohorts (i.e., separate exercise groups that ran at different times during the trial). Video recordings were collected by setting the camera up in the gymnasium with the aim of maximizing the amount of exercise space captured. The video camera was unmanned, therefore the field of view remained constant throughout the exercise session. Videos were included in this study if the visual and auditory quality was sufficient for coding the content of the exercise session.

### 2.3 Exercise sessions

The 90-minute exercise sessions took place three times per week for 12 weeks and were led by one of three physical therapists with additional clinical staff from The Hospital for Sick Children. The program was designed to begin with a warm-up that gradually increased heart rate, followed by a combination of aerobic training activities (e.g., circuit training, obstacle courses), games (e.g., tag, relay races), and/or sports (e.g., ball hockey, soccer), and ended with a cool-down activity. Participants wore heart rate monitors during the exercise sessions that provided intermittent visual feedback on exercise intensity (16). The activities completed in each session were not documented but exercise sessions were video recorded.

### 2.4 Process

The Exercise Session Content Log [adapted from Hilderley et al.'s (36) physical therapy intervention log where the physical therapist identifies the foci of an exercise from a standardized list] was developed to systematically code the physical and cognitive content of the exercise session videos. Instructions accompanied the log to promote consistent coding practices within and between raters. To use the log, the rater documented each exercise observed in the video (where a single exercise was defined as a physical activity with an obvious beginning and end).

#### 2.4.1 Physical fitness component rating

While watching each video, the rater used a checkmark system to indicate the approximate amount that each physical fitness component (cardiovascular, strength, coordination, speed, agility, balance, object control, flexibility) was present during an exercise, where one checkmark indicated a “low” amount (i.e., present for approximately 1%–24% of exercise duration), two checkmarks indicated a “moderate” amount (i.e., present for approximately 25%–74% of the exercise), and three checkmarks indicated a “high” amount (i.e., present for approximately ≥75% of the exercise). Ratings were based on what most participants were doing (i.e., a group-based approach). Physical fitness component definitions and examples were provided in the Exercise Session Content Log instructions (Table 1). The physical fitness components included on the Exercise Session Content Log were selected from Caspersen et al.'s (17) components of physical fitness, Lämmle et al.'s (20) two-level model of motor performance ability, and Lubans et al.'s (37) fundamental sport-specific movement skills. Items from these resources were omitted if they were not relevant to the context of this study (e.g., body composition) or combined if they were too difficult to differentiate between with video observation (e.g., strength = muscular endurance + power; cardiovascular = cardiorespiratory endurance + muscular endurance). Object control is a fundamental movement skill that was only mentioned by Lubans et al. (37) and was ultimately included because a coordination category alone would not distinguish between coordination content that did or did not involve an external object (e.g., ball, hockey stick, bean bag, scooter board).

**Table 1:** Definitions of the physical fitness components within the context of the current study.

Physical fitness component	Working definition	Examples
Cardiovascular	Continual use of large muscle groups aimed at elevating the heart rate.	What IS cardiovascular? Walking, running, playing soccer, circuit training, skipping rope, jumping jacks
		What IS NOT cardiovascular? Stretching, bicep curls, throwing a ball, balancing on one foot
Strength	Moving a muscle or body part against resistance. While resistance can involve weights, it can also involve moving the body weight against gravity and/or sustaining a position.	What IS strength? Squats, wall-sits, lunges, push-ups, sit ups, planks
		What IS NOT strength? Stretching, throwing a ball, balancing on one foot
Coordination^a^	Timing the use of body parts to perform a movement of appropriate speed, quality, direction, and distance.	What IS coordination? Jumping jacks, hitting a baseball, throwing a ball, skipping rope, changing directions while running
		What IS NOT coordination? Balancing on one foot, push ups
Speed	Movement that focuses on the rate that someone moves their entire body.	What IS NOT speed? Stretching, throwing a ball, balancing on one foot
		What IS speed? Running, tag, any activity with a timed component
Agility^a^	A rapid, whole body movement that involves a change in velocity or direction (54)	What IS agility? Avoiding a ball in dodgeball, changing directions to return a ball in tennis
		What IS NOT agility? A running race, push ups, yoga, throwing a ball
Object control^a^	Skilled use of external objects during an activity.	What IS object control? Kicking a ball, hitting a birdie with a badminton racquet, passing a ball with a hockey stick, hula hooping
		What IS NOT object control? Running around pylons, stepping over obstacles
Balance	Maintaining centre of mass within a reduced base of support during isolated activities (Note: This concept was modified to focus on isolated high level balance activities rather than the dynamic balance required when walking or running)	What IS balance? Tandem walking along a beam, standing/hopping on one foot
		What IS NOT balance? A running race, push ups, throwing a ball
Flexibility	Active or passive movement of a body part for the purpose of increasing the amount of movement at a joint.	What IS flexibility? Hamstring stretch, arm circles, leg swings, yoga?
		What IS NOT flexibility? Kicking a ball in soccer, jumping jacks

a Object control and agility involve aspects of coordination.

#### 2.4.2 Cognitive demand rating

Each exercise was assigned a cognitive demand rating of low (e.g., completing exercises in a circuit), medium (e.g., completing exercises in a relay race), or high (e.g., playing a game of floor hockey). This study-specific rating process was adapted from a scale that Heilmann et al. (38) used in their systematic review of executive function in open and closed-skill sports. Our rating system was based on whether the exercise was: (1) self-paced or externally paced, and (2) individual or team based. Within the context of this study, “self-paced” meant that the time to complete the exercise was not influenced by activity rules or instructions and there was no competitive element to influence the child's performance. Examples of self-paced exercises include jogging around the gym in warm-up, moving through an obstacle course one child after the other, or performing a hamstring stretch. In contrast, “externally paced” exercises meant that rules or instructions required the child to interact with other participants (e.g., side shuffling across the gym while passing a basketball back and forth with a partner) and/or maximize their performance relative to other participants (e.g., competing in a race).

Within the context of this study “individual” meant that participants completed the exercise independently within the group setting, while “team based” required participants to interact during the exercise to achieve a goal. If a team-based exercise did not involve interaction amongst team members, the exercise was considered “individual”. This decision was determined by listening to the instructions provided to the children on the video and observing the children during the exercise. The resultant cognitive demand rating system was a three-point scale where a score of 1 or “low” was an individual, self-paced exercise (e.g., jumping jacks during a circuit, skipping around the gym during warm-up), 2 or “medium” was an individual, externally paced exercise (e.g., jumping jacks during a team relay race, playing tag), and 3 or “high” was team-based, externally paced (e.g., soccer, ball hockey). Table 2 provides an example of a completed Exercise Session Content Log and Table 3 details the rationale for the physical fitness component and cognitive demand ratings associated with the example.

**Table 2:** Example of a completed Exercise Session Content Log.

Exercise	Start/Stop (video times)	Duration (minutes)	Physical fitness components								Cognitive demand rating
			Cardiovascular	Strength	Coordination	Speed	Agility	Balance	Object Control	Flexibility	Low = 1
											Med = 2
											High = 3
Warmup: follow the leader jogging, side shuffle, walking lunges, skipping around gym	1:00–9:30	8.5	✓✓✓	✓	✓			✓			1
Relay race: 5 squats, 2-foot side-to-side hop along/over a line, dribble basketball through pylons, run back to tag teammate; rest of team does jumping jacks while waiting	12:00–17:00, 18:00–24:30	11.5	✓✓	✓	✓	✓✓	✓		✓		2
Floor hockey: used two balls	25:00–39:00	14	✓✓		✓✓	✓	✓		✓✓		3
Freeze tag: 10 jumping jacks to unfreeze	40:30–50:30	10	✓✓✓		✓✓	✓✓	✓✓				2
Cooldown: walking around gym, stretches	51:00–59:00	8	✓							✓✓✓	1

Physical fitness components: low (✓)- present for 1%–24% of the exercise; moderate (✓✓)- present for 25%–74% of the exercise; high (✓✓✓)- present for ≥75% of the exercise. Cognitive rating scale: low- individual, internally paced; medium- individual, externally paced; high- group, externally paced.

**Table 3:** Example of the process for identifying the physical fitness components and assigning a cognitive demand rating for each exercise.

Exercise	Physical fitness components^a^			Cognitive demand rating and rationale
	Identification	Rating	Rationale	
Warmup: follow the leader	Jogging around gym: CV Side shuffle: CV, CO Walking lunges: BA, CV, ST Skipping around gym: CV, CO	CV = 3	Present continuously throughout entire exercise, 100% of exercise time	Rating: Low Individual activity without pacing demands
		CO = 1	Present continuously in 2 of 4 elements of exercise, approx. 20% of total exercise time	
		ST = 1	Present for 1 of 4 elements, approx. 10% of total exercise time	
		BA = 1	Present for 1 of 4 elements, approx. 10% of total exercise time	
Relay race	Squats: CV, SP, ST Two-footed side-to-side jump over line: AG, CV, CO, SP Dribbling basketball through pylons: AG, CV, CO, OB, SP Running back to the start to tag teammate: CV, SP Jumping jacks while waiting turn: CV, CO	CV = 3	Present continuously throughout entire exercise time	Rating: Medium Individual actions within each team with external pacing due to competition between two teams
		CO = 2	Present continuously in 3 of 5 elements, approx. 50% of total exercise time	
		SP = 2	Present in 4 of 5 elements, approx. 60% of total exercise time	
		AG = 1	Present intermittently in 2 of 5 elements, approx. 20% of total exercise time	
		OC = 1	Present continuously in 1 of 5 elements, approximately 20% of total exercise time	
		ST = 1	Present continuously in 1 of 5 exercises, approx. 10% of total exercise time	
Floor hockey	Running after two balls: CV, SP Changing directions to go after the ball: CV, AG Shooting and stick handling: CO, OB Playing goalie: AG, CO	CV = 2	Present intermittently throughout, approx. 60% of total exercise time	Rating: High Team interaction with external pacing due to competition between two teams
		CO = 2	Present intermittently throughout, approx. 60% of total exercise time	
		SP = 1	Present intermittently throughout, approx. 20% of total exercise time	
		AG = 1	Present occasionally throughout, approx. 10% of total exercise time	
		OC = 2	Present continuously for most of exercise, approx. 60% of total	
Freeze tag	Running: CV, SP Changing directions to tag or avoid being tagged: AG, CO, CV, SP 10 jumping jacks to “unfreeze” after tagged: CV, CO	CV = 3	Present continuously throughout, approx. 90% of total exercise	Rating: Medium Individual actions with external pacing due to competition with other children in the group
		CO = 2	Present intermittently throughout, approx. 40% of total exercise	
		SP = 2	Present continuously for approx. 50% of total exercise time	
		AG = 2	Present intermittently throughout for approx. 30% of total exercise time	
Cooldown	Walking around gym: CV	CV = 1	Present for 1 of 8 min of exercise	Rating: Low Individual activity without pacing
	Static stretches: FL	FL = 3	Present for 6 of 8 min of exercise	

a AG, agility; BA, balance; CV, cardiovascular; CO, coordination; FL, flexibility; OC, object control; SP, speed; ST, strength.

### 2.5 Interrater reliability

The primary rater (first author), who rated all 50 videos, is a physical therapist with extensive clinical experience working with CTBT. To establish consensus in the rating process and ensure that rating could be replicated regardless of rater expertise, secondary raters without clinical experience were trained by the first author to rate the videos using the Exercise Session Content Log. This process involved rating two practice videos and meeting after each video to compare scores, discuss discrepancies, come to a consensus on rating process, and update the rating manual. For inter-rater reliability (IRR), the first author and one secondary rater independently rated the same five videos using the Exercise Session Content Log. The IRR of physical fitness component and cognitive demand ratings were measured using a weighted kappa statistic to take the magnitude of rater disagreement into consideration when there are three or more rater categories (39). A target weighted kappa >0.80, which is considered almost perfect agreement (40), across the five videos was set as the threshold to surpass before the first author proceeded with rating the remaining study videos without a second rater performing duplicate ratings. Once the first author had completed all 50 video ratings, five additional videos were randomly selected and rated by a different secondary rater who had received the same training. IRR was then calculated between the 10 secondary rater videos and the corresponding primary rater videos to estimate IRR across the study timeline.

### 2.6 Analyses

Descriptive statistics were calculated using R statistical software (41). The Shapiro–Wilks Test was used to evaluate data normality, and based on the distribution, averages were reported as means (normal distribution) or medians (non-normal distribution) (42). Averages were calculated from the Exercise Session Content Log data: session duration (duration from the start of the first exercise to the end of the final exercise), individual exercise duration, time spent actively participating in exercise (sum of individual exercise durations), number of exercises per session, and time spent at each level of cognitive demand per session.

To address objective 1 (exercise categories observed), the average time spent on each exercise category (warm-up, aerobic training, games, sports, cool-down) was calculated. A descriptive summary of exercises frequently observed and common exercise variations/modifications was created. The average physical fitness component ratings were calculated by exercise category. Within each exercise category, the proportion of activities (across all sessions) for each cognitive demand rating was calculated. Inter-session variability in the proportion of time spent on exercises with different cognitive demand ratings in each session was presented visually.

To address objective 2 (physical fitness components observed), the time spent on each physical fitness component (cardiovascular, coordination, speed, agility, object control, strength, balance, flexibility) per session was calculated for low, moderate, high, and combined (low + moderate + high) ratings. These amounts were then divided by the total duration of all exercises in the session, and the average was taken across all sessions.

To address objective 3 (cognitive demand ratings of the exercises), exercises were divided into their cognitive demand rating categories (i.e., low, medium, or high). Within each cognitive demand category, the median physical fitness component rating (where low = 1, moderate = 2, and high = 3) was calculated for each physical fitness component (cardiovascular, coordination, speed, agility, object control, strength, balance, flexibility).

## 3 Results

### 3.1 Interrater reliability

Across the 10 videos rated by two independent raters, there were 68 exercises with nine observational categories for each exercise. The IRR was 0.85 (95% confidence interval [CI]: 0.80–0.90) for the first five videos and 0.89 (95% CI: 0.87–0.92) for all 10 videos.

### 3.2 Video summary

**Figure 1:**
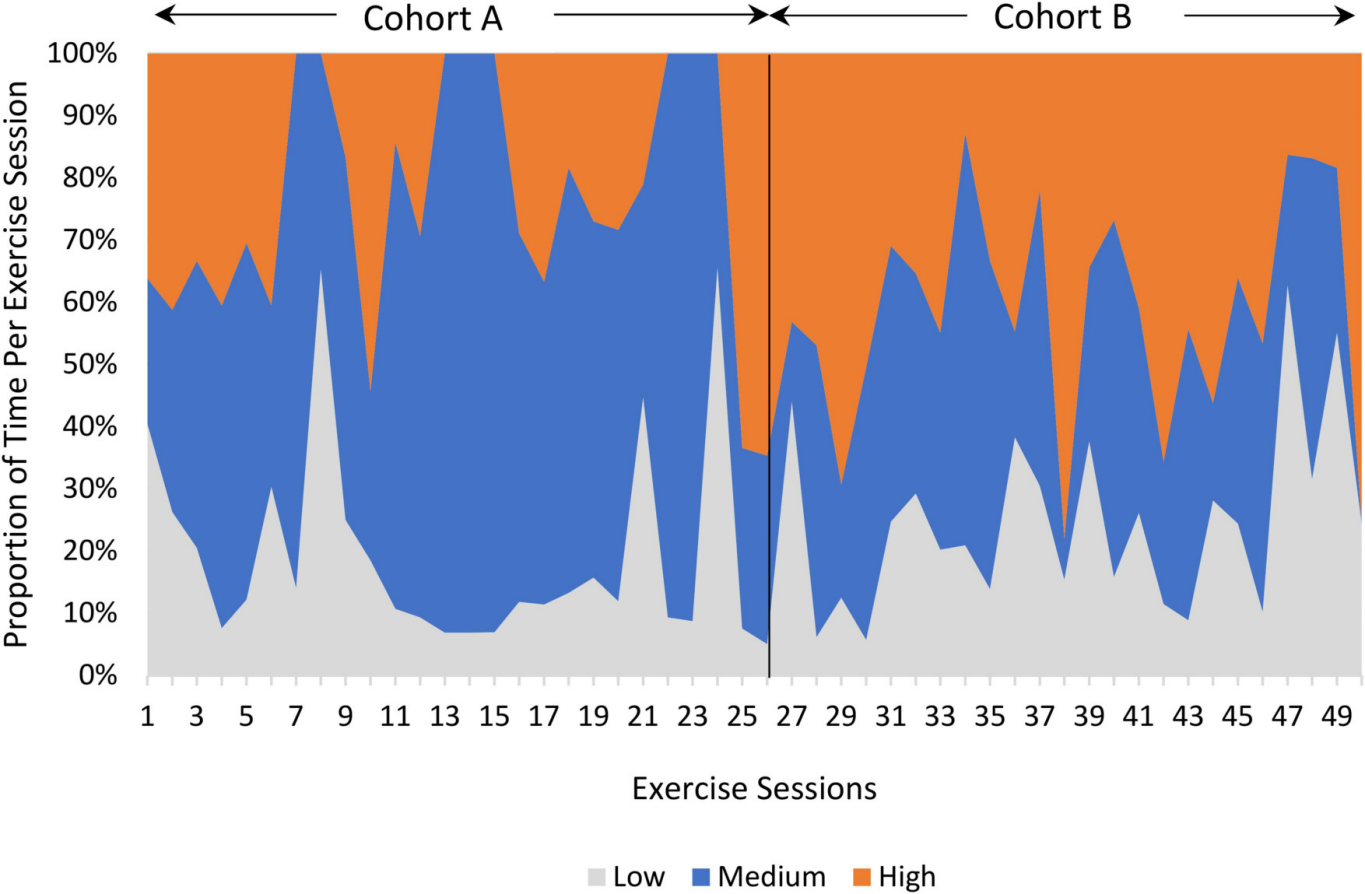
There was variability in the proportion of time per session spent on high (orange), medium (blue), and low (grey) cognitive demand activities across exercise sessions, with cohort A spending more time per session on activities with medium cognitive demand (i.e., games) and Cohort B spending more time per session on activities with high cognitive demand (i.e., sports).

Of 54 available videos, 50 videos were analyzed. These videos were distributed across two study cohorts (26 videos from cohort A, 24 videos from cohort B) and included videos from the beginning, middle, and end of each cohort. Four videos could not be analyzed due to poor video quality (no audio and/or the camera angle did not sufficiently capture the exercises). There were no missing/incomplete segments within the 50 analyzed videos. As not all data was normally distributed, medians were calculated instead of means. The median session duration was 64.0 min (interquartile range [IQR]: 8.5) with participants actively involved in exercise for 43.0 min (IQR: 7.5), with a median 7 exercises (IQR: 2) per session. The median proportion of time per session spent on activities with low, medium, and high cognitive demand ratings was 15.8% (IQR: 19.5), 44.1% (IQR: 26.8), and 33.3% (IQR: 28.3), respectively, with variability noted across exercise sessions and between study cohorts (Figure 1).

### 3.3 Objective 1- exercise categories observed in the exercise sessions

**Figure 2:**
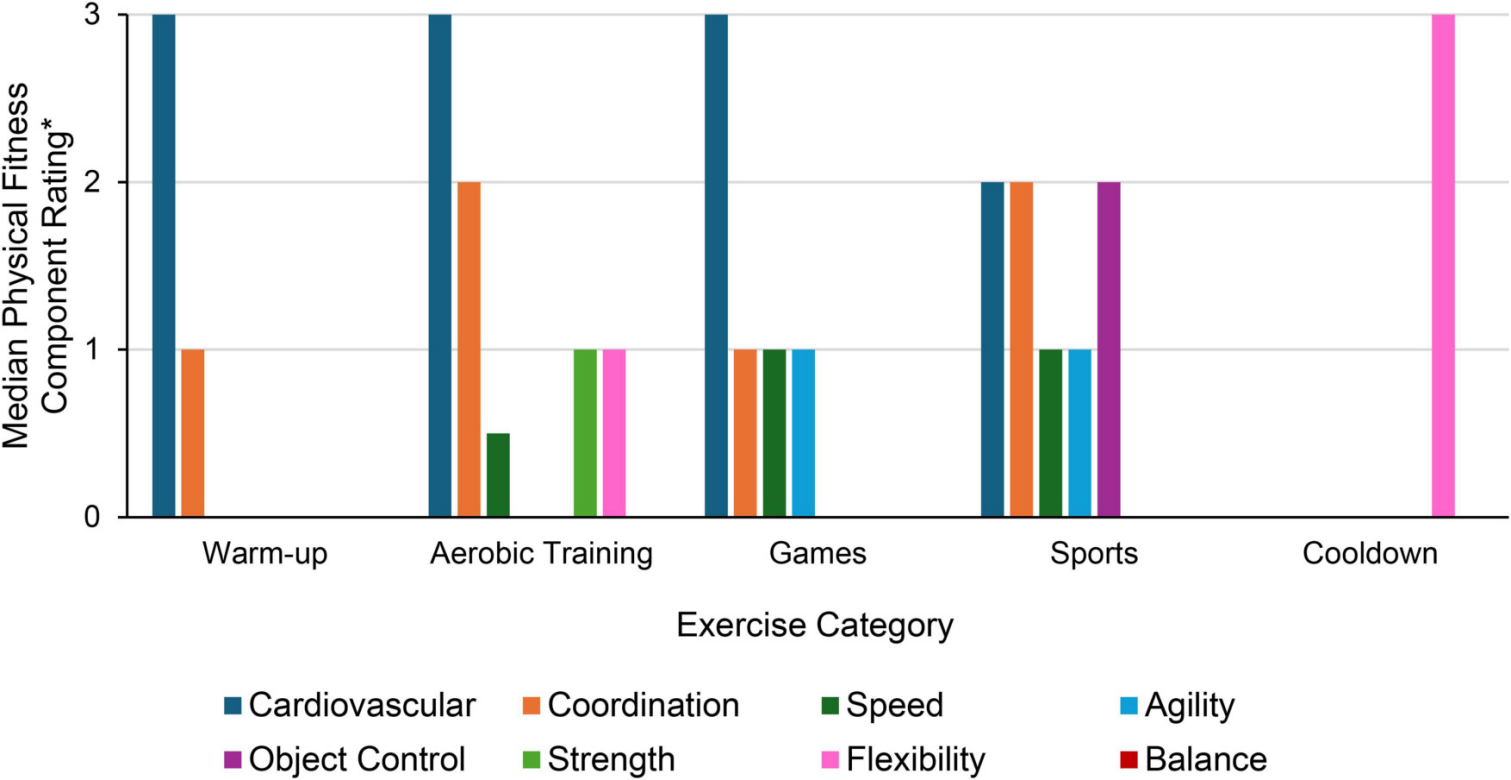
Cardiovascular content (blue) was the leading physical fitness component in the warm-up, aerobic training, and games categories, occurring in high amounts. For sports, there were three leading physical fitness components that occurred in moderate amounts: cardiovascular, coordination (orange), and object control (magenta) content. Flexibility (pink) was the leading physical fitness component in the cool-down category, occurring in high amounts (Other physical fitness component colors: speed = forest green; agility = light blue; strength = light green; balance = red). *Physical fitness component rating: 1 = low, 2 = moderate, 3 = high.

**Figure 3:**
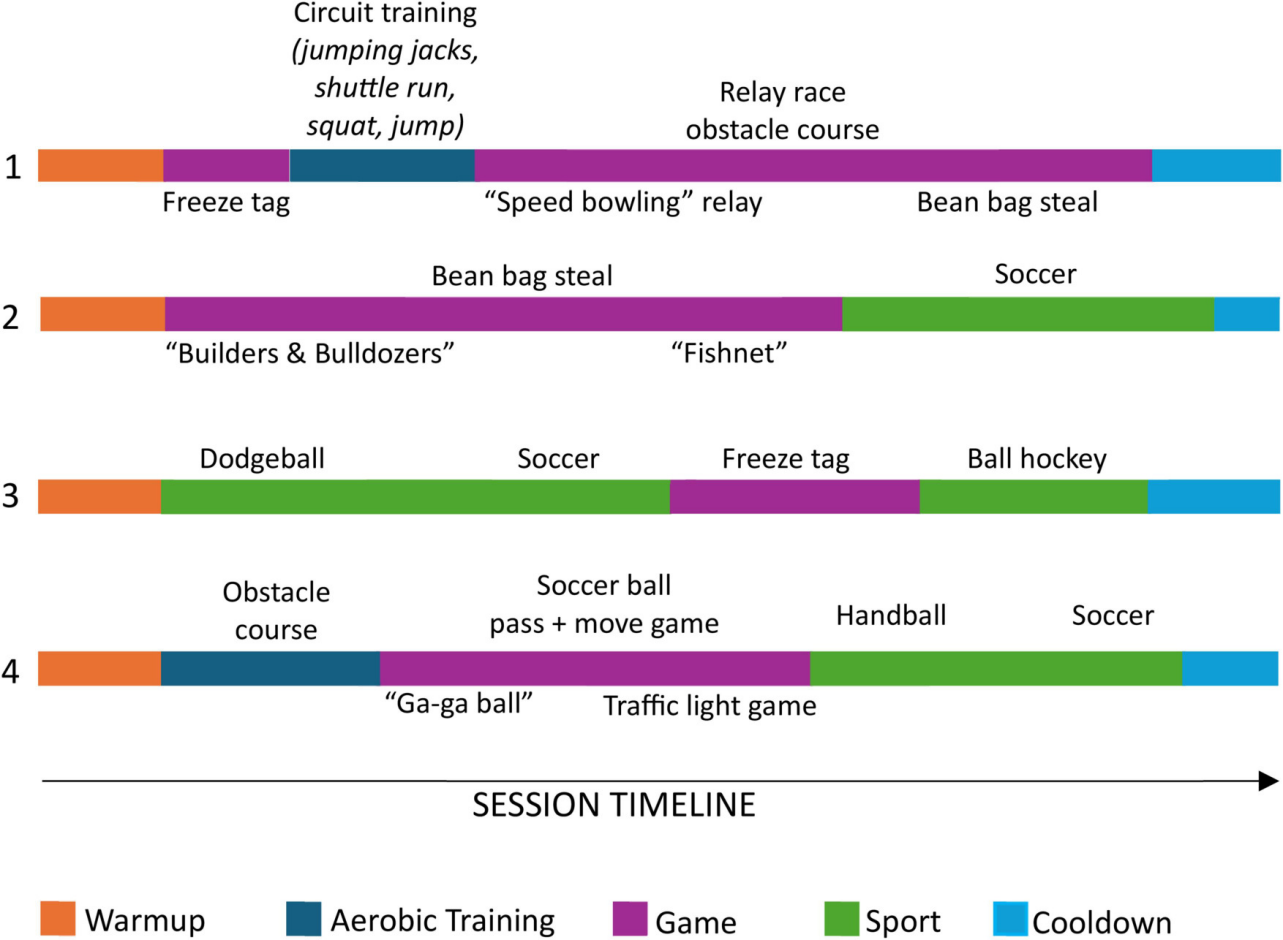
The timelines from four exercise sessions (1 and 2 are from cohort A, 3 and 4 are from cohort B) demonstrate the variability in exercise order and category across sessions. (The size of each category segment represents the proportion of the session spent on that category.)

The median time per session spent on each exercise category was: games- 17.0 min (IQR: 11.6), sports- 14.8 min (IQR: 11.9), aerobic training- 7.5 min (IQR: 11.0), warm-up- 5.5 min (IQR: 3.8), cool-down- 3.5 min IQR: 2.0). Across all sessions, the exercise categories with predominantly low cognitive demand ratings were cool-down (93.4% of the cool-down activities), warm-up (69.4% of the warm-up activities), and aerobic training (55.0% of the aerobic training activities). When exercises in these three categories received higher cognitive demand ratings, it was related to instructors adding additional requirements. For example, a warm-up exercise early in the program involved a participant telling the group their name before coming up with a warm-up exercise for the group to perform. They then pointed to another participant who had to recall the previous participant's name and create a new exercise. Games typically had medium cognitive demand ratings (90.4% of the games) and sports had high cognitive demand ratings (98.4% of the sports). When evaluating the physical fitness components by exercise category, cardiovascular content rated highest in the warm-up, aerobic training, and games categories (medians: 3, IQRs: 1,0,1 respectively) (Figure 2). In the sports category, cardiovascular, coordination, and object control achieved the highest ratings (medians: 2; IQRs: 0). In the cool-down category, flexibility was the highest rated physical fitness component (median: 3; IQR: 1.5). The order and type of aerobic training, games, and sports occurred varied across exercise sessions (see Figure 3 for examples).

Of the 62 sports, the most frequently observed were dodgeball (41.9%), soccer (24.2%), and ball hockey (22.6%). Sports were often modified (e.g., isolated to half the gymnasium, using two or more balls, goalies not being used, having to perform jumping jacks when hit with the dodgeball before resuming play). Of 131 games, the most frequently observed were variations of tag such as freeze tag, octopus, fishnet (27.5%), team-based territory games such as capture the flag or bean bag stealing (13.0%), and relay races with varying components such as dribbling a basketball, squats, hopping on one foot, jumping side to side over a line (7.6%). Modifications to game rules were also observed. For example, children completed a set of squats to be “unfrozen” in freeze tag, they jogged on the spot while waiting their turn in relay races, and they were encouraged to jog instead of walk during musical chair adaptations. Table 4 provides descriptions of exercises frequently observed in the aerobic training, games, and sports categories.

**Table 4:** Examples of aerobic training, games, and sports exercises.

Exercise category	Exercise name	Exercise description
Aerobic training	Animal walks	Across gym (e.g., crab walk, kangaroo hop, bear walk)
	Basketball skills	Shooting baskets with a partner. When you are shooting, your partner runs to retrieve the ball. When you miss, you switch roles
	Circuit	Rotate between stations: shuttle run, zig zag jumping forward along a line, skipping rope 10 times, lunges with dumbbells 10 times, shoot basketball 5 times, prone to stand 5 times
	Obstacle Course	One at a time, with a child starting once child ahead of them finished the first activity (repeat entire course once finished): weave through pylons, kick ball on net, bean bag toss, bicep curls, walk across bench and step over pylons, fast feet through hula hoop, kick ball on net, hop on one foot
	Scooter board skills	Moving across the gym on scooter board in different ways (e.g., on stomach propelling only with hands or with hands and feet, while sitting going forwards, backwards, sideways)
	Side shuffle while passing ball to partner	Start at one end of gym face to face with partner; chest pass ball back and forth until they reach the other side; repeat in other direction
	Snake	Walk/run around gym while following the directions the instructor moves in
	Traffic light	Run around gym. Perform different actions depending on what instructor calls out (e.g., red light = stop, green light = go, speedbump = jump, breakdown = lie down on back and wave arms and legs in the air)
Games	Bean bag steal	Two teams with pile of bean bags in the middle of the gym. Each team grabs beanbags (one at a time) and runs them back to their side of the gym (places on floor inside a hula hoop). The game is over when there are no more bean bags left in the middle. Team with most bean bags wins.
	Builders and bulldozers	Two teams (builders and bulldozers). Builders run around gym setting bowling pins on the ground, bulldozers run around knocking them down.
	Capture the beanbag	Like capture the flag but with multiple bean bags
	Flag tag	Multiple people “it”. To tag a person, need to pull flag off their belt. To earn the flag back, the person must do an exercise the instructor chooses (e.g., jumping jacks, push-ups, burpees, squats)
	Freeze tag	Multiple people “it”. When tagged, do 10 jumping jacks/squats/high knees to “unfreeze” and continue playing.
	Musical pylons	Jogging around gym while music playing. Run to a pylon when music stops. Child who doesn’t get to a pylon does an exercise to rejoin the game (e.g., lunges, burpees, high knees)
	Relay race	Two obstacle courses set up side-by-side. Teams race to finish first. While one child goes through the course, the team performs continuous movement (e.g., jogging on the spot, jumping jacks). Once you finish the obstacle course, you run back to tag the next person in line.
Sports	Ball hockey	Play with 2 balls
	Basketball	Modified so that children don’t need to dribble the ball. Instead, they take up to three steps before a pass. Need to make 3 passes before shooting at basket.
	Dodgeball	When hit, must complete an exercise (e.g., 10 jumping jacks) to rejoin the game
	Handball	On scooter boards; play with 2 balls
	Soccer	Play with 2 balls

### 3.4 Objective 2- physical fitness components within the exercises

**Figure 4:**
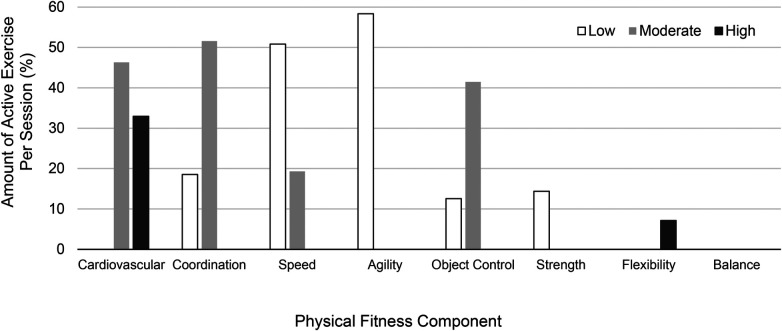
Median amounts of physical fitness components per exercise session (where low = present for ∼1%–24% of exercise duration, moderate = present for ∼25%–74% of the exercise, high = present for ≥75% of the exercise). Cardiovascular content was the leading physical fitness component present in high amounts (black). Coordination was the leading physical fitness component present in moderate amounts (gray), followed closely by cardiovascular and object control content. Agility content was the leading physical fitness component present in low amounts (white), followed closely by speed.

When low, moderate, and high physical fitness component ratings were combined, the median proportion of active exercise time per session involving cardiovascular content was 92.4% (IQR: 5.2%), followed by coordination (83.9%, IQR: 29.3%), speed (79.7%, IQR: 27.8%), agility (67.0%, IQR: 29.0%), object control (61.2%, IQR: 27.9%), strength (17.2%, IQR: 25.4%), flexibility (9.5%, IQR: 9.0%), and balance (0%, IQR: 1.9%). Notably, there were substantial differences between study cohorts for cardiovascular (cohort A: 93.18%, IQR 4.2%; cohort B: 86.7%, IQR 13.8%), coordination (cohort A: 66.9%, IQR: 39.0%; cohort B: 92.2%, IQR: 12.8%), speed (cohort A: 85.8%, IQR: 18.6%; cohort B: 64.9%, IQR: 28.7%), and object control (cohort A: 46.0%, IQR: 30.8%; cohort B: 68.2%, IQR: 15.7%) content. Figure 4 details the median proportion of active exercise time per session for each physical fitness component when rating categories were divided into low, moderate, and high amounts.

### 3.5 Objective 3- cognitive demand ratings of the exercises

**Figure 5:**
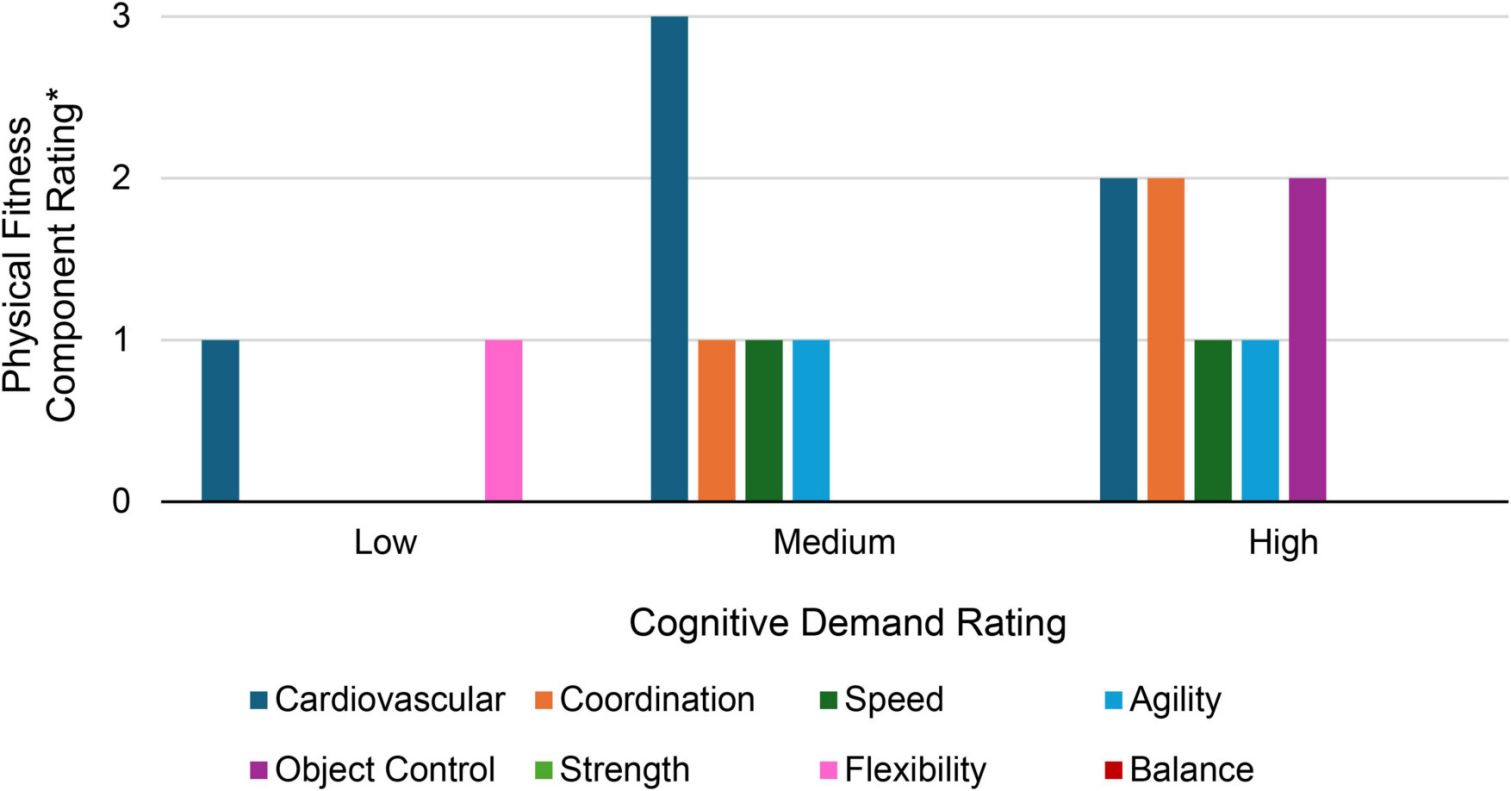
Median physical fitness component rating by cognitive demand rating. The amount of cardiovascular (blue) content varied across the different levels of cognitive demand. Medium cognitive demand exercises contained the highest amount of cardiovascular content. While the amount of speed (forest green), and agility (light blue) content remained the same between medium and high cognitive demand exercises, the amount of cardiovascular content decreased from medium to high cognitive demand and the amount of coordination (orange) and object control (magenta) content increased (Other physical fitness component colors: flexibility = pink; strength = light green; balance = red). *Physical fitness component rating: 1 = low, 2 = moderate, 3 = high.

The amount of each physical fitness component varied by cognitive demand rating category (i.e., low, medium, or high) (Figure 5). Cardiovascular content was higher for exercises with medium cognitive demand (median: 3.0, IQR: 1.0) than exercises with the high cognitive demand (median: 2.0, IQR: 0). In contrast, coordination and object control content were greater in exercises with high cognitive demand (medians: 2.0, IQRs: 0) than exercises with medium cognitive demand (medians: 1.0 and 0, respectively; IQRs: 1.0). For the low cognitive demand category, cardiovascular and flexibility content was the greatest (medians: 1.0; IQRs: 3.0).

## 4 Discussion

This study used video content analysis to describe the characteristics of our group exercise program and identify preliminary links between exercise categories, physical fitness components, and cognitive demand ratings in our group exercise program for CTBT. A predominant feature of our program was the combination of activities with high cardiovascular content/medium cognitive demand and activities with medium cardiovascular content/high cognitive demand within exercise sessions. Additionally, increased coordination content, particularly object control, was observed in activities with increased cognitive demand. Below we detail these observations, provide recommendations for future group exercise programs for CTBT in research and clinical/community settings, and discuss how our study limitations can be addressed in future research.

Cardiovascular content was the predominant physical fitness component observed in the program, which aligns with the emphasis on aerobic activity in the program design. However, the cardiovascular content was higher in exercises with medium cognitive demand ratings compared to exercises with high cognitive demand ratings, which could either indicate that the exercises in the high cognitive demand category did not intrinsically promote continual whole-body movement throughout the exercise (i.e., our rating criteria for a high cardiovascular content) or that the participants were unable to maintain continual whole-body movement during high cognitive demand exercises. Soccer and ball hockey were examples of high cognitive demand exercises frequently observed in the videos. While there are innate aspects of these sports that could decrease continual movement (e.g., being stationary as a goalkeeper or a player waiting for a pass), our exercise program adapted the typical rules of play to encourage continual movement across participants (e.g., multiple balls, no goalkeepers). Despite these adaptations, most participants did not maintain as much continual movement throughout the exercise compared to aerobic training activities and games. This finding suggests that a trade-off between exercise with high cardiovascular content and high cognitive demands may exist in CTBT. This potential trade-off aligns with dual-tasking research where simultaneously completing physical and cognitive tasks leads to decreased postural stability and motor control in healthy children and adults, as well as neurological populations (43–49). Ghanbarzadeh et al. (45) demonstrated that the negative impact of the cognitive load during a physical task is magnified in typically developing children compared to healthy adults, particularly as the complexity of the physical task increases. These dual-tasking costs are further exaggerated when comparing typically developing children to youth with traumatic brain injury, where youth with brain injury walk with decreased speed and increased step variability (48, 49). These findings support the hypothesis that CTBT may have more difficulty maintaining continuous movement during sports-based physical activities due to a combination of the inherent complexity of sports (e.g., rules, object control requirements, interaction with teammates and opponents) and their cognitive and physical challenges. However, it is unknown whether medium cognitive demand, high cardiovascular activities are more, less, or similarly beneficial for CTBT compared to high cognitive demand, moderate cardiovascular activities.

The extent that “secondary” physical fitness components (i.e., coordination, speed, agility, object control, strength, balance, flexibility) were part of the exercise program was unknown at the outset of this study, and the impact of different physical fitness components on cognitive demand was unclear. Our analysis indicated that the predominant secondary physical fitness component that occurred in moderate amounts was coordination, followed by object control, while agility was the predominant physical fitness component that occurred in low amounts, followed by speed. Prior research indicates that exercise involving the coordination of complex motor skills has increased cognitive demands which leads to improvements in executive function (30, 31). Our observations supported this connection between coordination content and cognitive demands, with increased coordination content observed in exercises with higher cognitive demand ratings. Exercises with high cognitive demand ratings had increased coordination and object control content compared to exercises with moderate cognitive demand ratings. Although object control content ratings were not mutually exclusive from the coordination content ratings (as object control has considerable coordination requirements due to the need for precise timing and movement of specific body parts), its increased presence appeared to be a differentiating factor between exercises with medium and high cognitive demand ratings. Thus, the combined presence of coordination and object control content may contribute to the increased the cognitive demands of an exercise.

Maintaining cognitive engagement in exercise is integral to improving cognitive skills in children (30, 50–52). As CTBT often have difficulty sustaining their attention (2), an exercise program consisting solely of high cognitive demands may be too challenging and lead to decreased engagement and participation. Conversely, physical fatigue may prevent CTBT from sustaining constant cardiovascular activity with lower cognitive demands for an entire exercise session. Therefore, alternating between activities with high cognitive demands (with moderate cardiovascular content) and high cardiovascular content (with medium cognitive demands) may be an effective strategy for maximizing CTBT' participation in an exercise program that aims to improve cognition. The variability in exercise session content from session to session and across cohorts may have been due to instructors adjusting session content to optimize participant engagement.

### 4.1 Recommendations

We have several recommendations when running a group exercise program for CTBT in a clinical/community setting and/or designing clinical trials to evaluate exercise program effectiveness.

#### 4.1.1 For research settings

Given the differences between the proposed exercise session length (90 min) and the video length (median 64.0 min), exercise session duration should be documented in real-time and monitored in future trials/programs to ensure dose consistency. Similarly, the study participants spent considerably less time on aerobic training, warm-up, and cool-down than the intervention structure outlined. This discrepancy may reflect the instructors' deliberate efforts to select exercises that promoted participant engagement (i.e., they may have observed increased engagement during games/sports over aerobic training). To better understand, instructors' decisions, future programs should include instructor documentation of session activities and rationale for protocol deviations. Future research should also gather participant feedback regarding their enjoyment of different activities and compare engagement and cognitive outcomes between programs with different compositions of exercise categories (e.g., programs with and without aerobic training that is not categorized as a game or sport).

#### 4.1.2 For clinical/community settings

While this study did not determine the optimal combination of activities that should be included in a group exercise program for CTBT, it provides practical considerations for clinical and community settings. We recommend that exercise program instructors receive training about the potential differences in cognitive demand across different exercise categories, the possible impact of physical fitness components on cognitive demand (e.g., activities involving object control may be more cognitively demanding), and the potential trade-off between cardiovascular content and cognitive demand in CTBT, where youth may move less during activities with higher cognitive demands depending on their familiarity with the activity and their cognitive limitations. Instructors should also adjust the types of exercises within each exercise session to meet the needs of the participants in the group. These adjustments may involve increasing or decreasing the proportion of aerobic training, games, or sports depending on participant ability/interest/engagement. For example, if a group is not as motivated by competitive sports, the instructors could introduce cooperative games instead. Instructors should also consider how activities with high physical or high cognitive demand are presented. For example, to maintain active participation throughout the instructors could decide to intersperse aerobic training exercises (i.e., activities with high cardiovascular content and low cognitive demands) between sports (i.e., activities with medium cardiovascular content and high cognitive demand) rather than completing all aerobic training activities prior to introducing the sports. Finally, instructors should always plan and document session content, as this process promotes reflection upon activity selection, exercise duration, and successes/challenges.

### 4.2 Study limitations and future research directions

It is important to acknowledge the limitations of this study design—a descriptive design cannot be used to infer intervention efficacy, and content coding is susceptible to rater bias. While video observation is useful for analyzing complex interactions (53), analyses are limited by video quality. In this study, the unmanned video camera (i.e., its position and angle was not adjusted during the session) was positioned to capture as much of the exercise space as possible, which meant that participant movement that occurred at distance from the camera could not be analyzed with the same level of scrutiny as individuals positioned closer to the camera. This limitation combined with the subjectivity of content ratings, may have led to some content being overlooked and some content being overemphasized. While the variability in session content between the two study cohorts may truly represent differences in content, the limitations of video observation may have augmented these differences.

Further, video quality prevented us from being able to identify and analyze individual participants. Given the retrospective nature of this study and our inability to identify participants, we were unable to collect and analyze data for individual participants. Instead, the content ratings were based on observation of the group as whole, individual engagement and participation was not explored, and participant physical and cognitive outcomes could not be linked to individual content ratings. Future research should incorporate higher resolution videos and real-time session engagement ratings (self-reported and independent observation) along with heart rate monitoring and step counts (accelerometry) to permit data triangulation and in-depth analysis of how children's level of participation factors into their cognitive and physical outcome measure change scores.

While reproducible, our cognitive demand ratings were a highly simplified way of identifying the cognitive difficulty of an activity at the group level based on the properties of a physical activity. This rating acted as a proxy for cognitive engagement and did not consider the cognitive nuances (e.g., attention, working memory) of each activity, nor did it consider individual factors that influence cognitive demand such as age, physical ability, baseline cognitive function, and the participants' prior experience with the activity or motor skill. Factoring these covariates into future prospective research would help to understand if/how a group exercise program cognitively challenges individual participants and may help to more accurately differentiate between the cognitive demands of different exercises/activities. Additionally, the connection between increased coordination/object control content and the cognitive demand is descriptive rather than confirmatory.

The cardiovascular content ratings in this study were based on observation of whole-body movement which does not directly indicate the physiological intensity of cardiovascular activity. Unfortunately, the individual heart rate data collected during the exercise sessions was corrupted and could not be used to corroborate/refute our cardiovascular content ratings. Future research is required to explore the validity of our cardiovascular content ratings by evaluating the association between video observation and heart rate, accelerometry, and perceived exertion ratings at the individual level. Future exercise research in CTBT should also compare exercise programs with differing content (e.g., sport vs. aerobic training, strength vs. motor skills) to determine the individual and combined impact of aerobic, strength, balance, and motor skill training on cognitive and physical outcomes.

Video analysis permits repeated viewing of an event, which generally lends itself to higher inter-rater reliability because raters can practice and review coding procedures (53). Despite demonstrating good inter-rater reliability across the study timeline, there is still a possibility that observer bias influenced the session ratings because the first author was the primary rater for all 50 videos. As such, we recommend future studies follow a similar rater training procedure and then distribute the primary video rating equally among several raters.

## 5 Conclusion

The analysis of the physical fitness components within our group exercise program for CTBT and the cognitive demands associated with those exercises suggested a possible trade-off between cardiovascular content and cognitive demand within the exercises in this program where exercises with high cardiovascular content typically had medium cognitive demand ratings while exercises with medium cardiovascular content often had high cognitive demand ratings. While cardiovascular content was the foundation for the exercise program design, the presence of other physical fitness components appeared to influence the cognitive demands of each exercise. Increased coordination content was observed during exercises with increased cognitive demands, particularly when the coordination content involved object control. This study emphasizes the importance of characterizing the content of group exercise programs as a precursor to isolating the effective elements within a group exercise program for CTBT, identifies cognitive and physical features to consider when running a group exercise program for CTBT, and provides a process for isolating physical and cognitive elements in future clinical trials that intervention efficacy.

## Data Availability

The datasets presented in this article are not readily available because the data is not permitted to be released.

## References

[1] Nicklin E Velikova G Hulme C Rodriguez Lopez R Glaser A Kwok-Williams M Long-term issues and supportive care needs of adolescent and young adult childhood brain tumour survivors and their caregivers: a systematic review. Psychooncology. (2019) 28:477–87. 10.1002/pon.4989 30657618

[2] Oyefiade A Paltin I Luca D Hardy CR Grosshans KK Chintagumpala DR Cognitive risk in survivors of pediatric brain tumors. J Clin Oncol. (2021) 39:1718–26. 10.1200/JCO.20.02338 33886348 PMC8260914

[3] Piscione PJ Bouffet E Mabbott DJ Shams I Kulkarni AV. Physical functioning in pediatric survivors of childhood posterior fossa brain tumors. Neuro Oncol. (2014) 16:147–55. 10.1093/neuonc/not138 24305707 PMC3870837

[4] Gielis M Dirix V Vanderhenst E Uyttebroeck A Feys H Sleurs C Better detection of reduced motor functioning in brain tumor survivors based on objective motor assessments: an incentive for improved standardized follow-up. Eur J Pediatr. (2022) 181:2731–40. 10.1007/s00431-022-04472-1 35476292 PMC9192471

[5] Varedi M Lu L Phillips NS Partin RE Brinkman TM Armstrong GT Balance impairment in survivors of pediatric brain cancers: risk factors and associated physical limitations. J Cancer Surviv. (2021) 15:311–24. 10.1007/s11764-020-00932-5 32895869 PMC7936993

[6] King TZ Smith KM Ivanisevic M. The mediating role of visuospatial planning skills on adaptive function among young-adult survivors of childhood brain tumor. Arch Clin Neuropsychol. (2015) 30:394–403. 10.1093/arclin/acv033 26055499

[7] Law N Smith ML Greenberg M Bouffet E Taylor MD Laughlin S Executive function in paediatric medulloblastoma: the role of cerebrocerebellar connections. J Neuropsychol. (2017) 11:174–200. 10.1111/jnp.12082 26242813

[8] Robinson KE Fraley CE Pearson MM Kuttesch JF Compas BE. Neurocognitive late effects of pediatric brain tumors of the posterior fossa: a quantitative review. J Int Neuropsychol Soc. (2013) 19:44–53. 10.1017/S1355617712000987 23095276

[9] Khaleqi-Sohi M Sadria G Ghalibafian M Khademi-Kalantari K Irannejad S. The effects of physical activity and exercise therapy on pediatric brain tumor survivors: a systematic review. J Bodyw Mov Ther. (2022) 30:1–9. 10.1016/j.jbmt.2021.11.003 35500954

[10] Sharma B Allison D Tucker P Mabbott D Timmons BW. Exercise trials in pediatric brain tumor: a systematic review of randomized studies. J Pediatr Hematol Oncol. (2021) 43:59–67. 10.1097/MPH.0000000000001844 32604333

[11] Chaddock L Pontifex MB Hillman CH Kramer AF. A review of the relation of aerobic fitness and physical activity to brain structure and function in children. J Int Neuropsychol Soc. (2011) 17:975–85. 10.1017/S1355617711000567 22040896

[12] Chang YK Labban JD Gapin JI Etnier JL. The effects of acute exercise on cognitive performance: a meta-analysis. Brain Res. (2012) 1453:87–101. 10.1016/j.brainres.2012.02.068 22480735

[13] Chin LM Keyser RE Dsurney J Chan L. Improved cognitive performance following aerobic exercise training in people with traumatic brain injury. Arch Phys Med Rehabil. (2015) 96:754–9. 10.1016/j.apmr.2014.11.009 25433219 PMC4380661

[14] Christiansen L Beck MM Bilenberg N Wienecke J Astrup A Lundbye-Jensen J. Effects of exercise on cognitive performance in children and adolescents with ADHD: potential mechanisms and evidence-based recommendations. J Clin Med. (2019) 8:841. 10.3390/jcm8060841 31212854 PMC6617109

[15] Zheng G Zhou W Xia R Tao J Chen L. Aerobic exercises for cognition rehabilitation following stroke: a systematic review. J Stroke Cerebrovasc Dis. (2016) 25:2780–9. 10.1016/j.jstrokecerebrovasdis.2016.07.035 27554073

[16] Riggs L Piscione J Laughlin S Cunningham T Timmons BW Courneya KS Exercise training for neural recovery in a restricted sample of pediatric brain tumor survivors: a controlled clinical trial with crossover of training versus no training. Neuro Oncol. (2017) 19:440–50. 10.1093/neuonc/now177 27555603 PMC5464296

[17] Caspersen CA Powell KE Christenson GM. Physical activity, exercise, and physical fitness: definitions and distinctions for health-related research synopsis. Public Health Rep. (1985) 100:126–31. 3920711 PMC1424733

[18] Esteban-Cornejo I Tejero-González CM Martinez-Gomez D Del-Campo J González-Galo A Padilla-Moledo C Independent and combined influence of the components of physical fitness on academic performance in youth. J Pediatr. (2014) 165:306–12.e2. 10.1016/j.jpeds.2014.04.044 24952710

[19] Kolimechkov S. Physical fitness assessment in children and adolescents: a systematic review. Eur J Phys Educ Sport Sci. (2017) 3:65–79. 10.5281/zenodo.495725

[20] Lämmle L Tittlbach S Oberger J Worth A Bös K. A two-level model of motor performance ability. J Exerc Sci Fit. (2010) 8:41–9. 10.1016/S1728-869X(10)60006-8

[21] Schmidt M Egger F Benzing V Jäger K Conzelmann A Roebers CM Disentangling the relationship between children’s motor ability, executive function and academic achievement. PLoS One. (2017) 12:e0182845. 10.1371/journal.pone.0182845 28817625 PMC5560562

[22] Shu Y He Q Xie Y Zhang W Zhai S Wu T. Cognitive gains of aerobic exercise in patients with ischemic cerebrovascular disorder: a systematic review and meta-analysis. Front Cell Dev Biol. (2020) 8:582380. 10.3389/fcell.2020.582380 33392183 PMC7775417

[23] Singh AS Saliasi E Van DenBerg V Uijtdewilligen L Groot D Jolles RHM Effects of physical activity interventions on cognitive and academic performance in children and adolescents: a novel combination of a systematic review and recommendations from an expert panel. Br J Sports Med. (2019) 53:640–7. 10.1136/bjsports-2017-098136 30061304

[24] Young J Angevaren M Rusted J Tabet N. Aerobic exercise to improve cognitive function in older people without known cognitive impairment. Cochrane Database Syst Rev. (2015) 2015:1–118. 10.1002/14651858.CD005381.pub4 PMC10554155 25900537

[25] Feter N Alt R Dias MG Rombaldi AJ. How do different physical exercise parameters modulate brain-derived neurotrophic factor in healthy and non-healthy adults? A systematic review, meta-analysis and meta-regression. Sci Sports. (2019) 34(5):293–304. 10.1016/j.scispo.2019.02.001

[26] Ferrer-Uris B Ramos MA Busquets A Angulo-Barroso R. Can exercise shape your brain? A review of aerobic exercise effects on cognitive function and neuro-physiological underpinning mechanisms. AIMS Neurosci. (2022) 9:150–74. 10.3934/Neuroscience.2022009 35860684 PMC9256523

[27] Aghjayan SL Lesnovskaya A Esteban-Cornejo I Peven JC Stillman CM Erickson KI. Aerobic exercise, cardiorespiratory fitness, and the human hippocampus. Hippocampus. (2021) 31:817–44. 10.1002/hipo.23337 34101305 PMC8295234

[28] Firth J Stubbs B Vancampfort D Schuch F Lagopoulos J Rosenbaum S Effect of aerobic exercise on hippocampal volume in humans: a systematic review and meta-analysis. Neuroimage. (2018) 166:230–8. 10.1016/j.neuroimage.2017.11.007 29113943

[29] Szulc-Lerch KU Timmons BW Bouffet E Laughlin S de Medeiros CB Skocic J Repairing the brain with physical exercise: cortical thickness and brain volume increases in long-term pediatric brain tumor survivors in response to a structured exercise intervention. Neuroimage Clin. (2018) 18:972–85. 10.1016/j.nicl.2018.02.021 29876282 PMC5987848

[30] Best JR. Effects of physical activity on children’s executive function: contributions of experimental research on aerobic exercise. Dev Rev. (2010) 30:331–51. 10.1016/j.dr.2010.08.001 21818169 PMC3147174

[31] Pesce C. Shifting the focus from quantitative to qualitative exercise characteristics in exercise and cognition research. J Sport Exerc Psychol. (2012) 34:766–86. 10.1123/jsep.34.6.766 23204358

[32] Tomporowski PD McCullick B Pendleton DM Pesce C. Exercise and children’s cognition: the role of exercise characteristics and a place for metacognition. J Sport Health Sci. (2015) 4:47–55. 10.1016/j.jshs.2014.09.003

[33] Nejati V Derakhshan Z. The effect of physical activity with and without cognitive demand on the improvement of executive functions and behavioral symptoms in children with ADHD. Expert Rev Neurother. (2021) 21:607–14. 10.1080/14737175.2021.1912600 33849353

[34] Reimer T Park ES Hinsz VB. Shared and coordinated cognition in competitive and dynamic task environments: an information-processing perspective for team sports. Int J Sport Exerc Psychol. (2006) 4:376–400. 10.1080/1612197X.2006.9671804

[35] Singer RN. Performance and human factors: considerations about cognition and attention for self-paced and externally-paced events. Ergonomics. (2000) 43:1661–80. 10.1080/001401300750004078 11083145

[36] Hilderley AJ Fehlings D Lee GW Wright FV. Comparison of a robotic-assisted gait training program with a program of functional gait training for children with cerebral palsy: design and methods of a two group randomized controlled cross-over trial. Springerplus. (2016) 5:1–14. 10.1186/s40064-016-3535-0 27843743 PMC5084143

[37] Lubans DR Morgan PJ Cliff DP Barnett LM Okely AD. Fundamental movement skills in children and adolescents: review of associated health benefits. Sport Med. (2010) 40:1019–35. 10.2165/11536850-000000000-00000 21058749

[38] Heilmann F Weinberg H Wollny R. The impact of practicing open- vs. closed-skill sports on executive functions—a meta-analytic and systematic review with a focus on characteristics of sports. Brain Sci. (2022) 12:1071. 10.3390/brainsci12081071 36009134 PMC9406193

[39] Li M Gao Q Yu T. Kappa statistic considerations in evaluating inter-rater reliability between two raters: which, when and context matters. BMC Cancer. (2023) 23:1–5. 10.1186/s12885-023-11325-z 37626309 PMC10464133

[40] Landis J Koch G. The measurement of observer agreement for categorical data. Biometrics. (1977) 33:159–74. 10.2307/2529310 843571

[41] R Core Team. R: a language and environment for statistical computing. Vienna, Austria: R Foundation for Statistical Computing (2024).

[42] Portney LG. Foundations of Clinical Research: Applications to Evidence-Based Practice. 4th ed. Philadelphia, PA: F.A. Davis (2020).

[43] Bayot M Dujardin K Tard C Defebvre L Bonnet CT Allart E The interaction between cognition and motor control: a theoretical framework for dual-task interference effects on posture, gait initiation, gait and turning. Neurophysiol Clin. (2018) 48(6):361–75. 10.1016/j.neucli.2018.10.003 30487064

[44] Brown LA Hall EE Ketcham CJ Patel K Buckley TA Howell DR Turn characteristics during gait differ with and without a cognitive demand among college athletes. J Sport Rehabil. (2020) 29:448–53. 10.1123/jsr.2018-0129 30860425

[45] Ghanbarzadeh A Azadian E Majlesi M Jafarnezhadgero AA Akrami M. Effects of task demands on postural control in children of different ages: a cross-sectional study. Appl Sci. (2022) 12:113. 10.3390/app12010113

[46] Liparoti M. Effects of motor and cognitive loads on postural stability in healthy children. J Hum Sport Exerc. (2021) 16:S913–21. 10.14198/jhse.2021.16.Proc3.08

[47] Ghai S Ghai I Effenberg AO. Effects of dual tasks and dual-task training on postural stability: a systematic review and meta-analysis. Clin Interv Aging. (2017) 12:557–77. 10.2147/CIA.S125201 28356727 PMC5367902

[48] Abdul Rahman RA Rafi F Hanapiah FA Nikmat AW Ismail NA Manaf H. Effect of dual-task conditions on gait performance during timed up and go test in children with traumatic brain injury. Rehabil Res Pract. (2018) 2018:2071726. 10.1155/2018/2071726 30402290 PMC6193351

[49] Katz-Leurer M Rotem H Keren O Meyer S. Effect of concurrent cognitive tasks on gait features among children post-severe traumatic brain injury and typically-developed controls. Brain Inj. (2011) 25:581–6. 10.3109/02699052.2011.572943 21534735

[50] Mavilidi MF Ruiter M Schmidt M Okely AD Loyens S Chandler P A narrative review of school-based physical activity for enhancing cognition and learning: the importance of relevancy and integration. Front Psychol. (2018) 9:2079. 10.3389/fpsyg.2018.02079 30464752 PMC6234858

[51] Schmidt M Jäger K Egger F Roebers CM Conzelmann A. Cognitively engaging chronic physical activity, but not aerobic exercise, affects executive functions in primary school children: a group-randomized controlled trial. J Sport Exerc Psychol. (2015) 37:575–91. 10.1123/jsep.2015-0069 26866766

[52] Vazou S Pesce C Lakes K Smiley-Oyen A. More than one road leads to Rome: a narrative review and meta-analysis of physical activity intervention effects on cognition in youth. Int J Sport Exerc Psychol. (2019) 17:153–78. 10.1080/1612197X.2016.1223423 31289454 PMC6615761

[53] Asan O Montague E. Using video-based observation research methods in primary care health encounters to evaluate complex interactions. Inform Prim Care. (2014) 21:161–70. 10.14236/jhi.v21i4.72 25479346 PMC4350928

[54] Sheppard JM Young WB. Agility literature review: Classifications, training and testing. J Sport Sci. (2006) 24:9, 919–32. 10.1080/02640410500457109 16882626

